# Feasibility of point of care testing for prevention and management of breast cancer therapy associated comorbidities in 6 African countries: short communication

**DOI:** 10.1186/s13104-022-06204-y

**Published:** 2022-10-22

**Authors:** Jean Paul Muambngu Milambo, James Ndirangu, Peter S Nyasulu, John M Akudugu

**Affiliations:** 1 Department of Public health, Faculty of Medicine and Health Sciences, University of Free State, Florence Street 7530 Bellville, Cape Town, South Africa; 2 University of Stellenbosch, Stellenbosch, South Africa

**Keywords:** Aromatase inhibitors, Point of care testing, Postmenopausal breast cancer, Cardiovascular risk factors

## Abstract

**Objective:**

Obesity and mediators of inflammation have been identified as the most important risk and predictive factors in postmenopausal breast cancer (BC) survivors using aromatase inhibitors (AIs). This study was conducted to assess the impact of point of care technology (PCOT) as part of pathology supported genetic testing (PSGT) to prevent BC therapy-associated comorbidities in African settings.

**Results:**

The study revealed that high sensitivity C-reactive protein (hs-CRP) and body mass index (BMI) are predictors of cardiovascular (CVD) related adverse events in obese postmenopausal patients subjected to AIs. There were statistically significant variations in total body fat (TBF), weight, hs-CRP, body mass index (BMI), homocysteine, ferritin, and calcium between baseline and after 24 months of follow-up. The above inflammatory markers can be incorporated in pathology supported genetic testing (PSGT) using HyBeacon® probe technology at POC for prediction and management of AI-associated adverse events among postmenopausal breast cancer survivors and associated comorbidities. The barriers for implementation of POCT application among six African countries for diagnosis of breast cancer were documented as insufficient of BC diagnosis and management capacity at different levels of health system.

## Introduction

The finding that the 10-year predicted risk for cardiovascular disease (CVD) equals or exceeds recurrence risk of BC in postmenopausal patients, led to recommendations to offer chronic disease screening programs in cancer survivors[1, 2]. Aromatase inhibitors (AIs) are main treatment strategy for hormone receptor-positive BC patients. However, by decreasing levels of serum estrogens, they also potentially reduce the protective effect of estrogens on the CVD system[3, 4]. Co-morbidities associated with anti-cancer treatment adds to this complexity [4]. A study conducted on genetic testing services available through the National Health Laboratory Service, revealed a shortage of geneticists, counsellors and medical scientists trained to deliver personalized genomic medicine to the African public[5]. Since pathology tests, such as those used to assess hormone receptor status or high-sensitivity C-reactive protein (hs-CRP) levels may reflect obesity, this multi-gene assay currently performed using real-time polymerase chain reaction (PCR) methodology, combines pathology and genetic test results with lifestyle risk factors for clinical interpretation of the test results. Turn-around time using the pathology supported genetics testing (PSGT) approach is typically 1–2 weeks before the laboratory results are available [6]. Study conducted by Milambo et.al.(2021) on the efficacy of medically supervised exercises(MSE) in preventing aromatase inhibitors induced adverse events revealed that MSE can be used to improve range of motion(ROM) and heath related quality of life (HRQOL) in postmenopausal breast cancer patients and associated comorbidities [7]. A number of shared risk factors between postmenopausal status and CVD should be considered, while treating postmenopausal BCS. The results of meta-regression revealed that there was moderate evidence that medically supervised exercises (MSE) decreased the mean difference (MD) of inflammatory markers in BCS with CVD risk factors. Ideally, genetic testing should be performed at the point of care (POC) to reduce the time from sample collection to obtaining a result. While risk reduction intervention guided by a patient’s genotype may be associated with decreased medication side effects/comorbidities, the potential benefits of POC testing remains to the demonstrated in postmenopausal BC patients in resource-limited clinical settings of Africa where data of this kind is missing. This study was conducted to assess the impact of point of care technology (PCOT) as part of pathology supported genetic testing (PSGT) to prevent BC therapy-associated comorbidities in African settings.

## Methods and materials

We summarized the research findings from published PhD thesis using existing statistical analysis to identify the gaps reported in different chapters by the authors including the scoping review of published studies for evidence based communication for further studies to be conducted in African settings. The cross sectional study was conducted in 6 African countries to assess the level of breast cancer diagnosis, management pathways comparing to the current tools used in developed countries. The whole thesis is available at Stellenbosch University website: https://scholar.sun.ac.za/handle/10019.1/123642.

Study conducted by the authors included 126 breast cancer women from South African population using aromatase inhibitors (AIs) at their menopausal age.

Pathology-supported genetic testing (PSGT) algorithm aims at preventing/reversing disability for complex diseases (cancers, cardiovascular conditions, diabetes, cholesterol disorder and other metabolic syndromes…) PSGT is starting with a questionnaire-based risk assessment, including family history and lifestyle factors. Measurement of clinical profiles, biomedical and genetic predictors. POCT or PSGT is a component of interventional strategy based on the concept of gene-environment-lifestyle factors. The point of care technology (POCT) is defined as pathology supported genetic testing which includes assessment of clinical markers (BMI), biomedical markers (hs-CRP) and genetic markers to identify Aromatase inhibitors associated side effects during the period of time for breast cancer survivorship. The primary study conducted by the authors included 126 breast cancer women from South African population using aromatase inhibitors (AIs) at their menopausal age. These women were from colored population from Western Cape Province of South Africa. demographics data were published in the main studies [7, 10, 11]. The 6 African countries surveyed including DRC, Burundi, South Africa, Eswatini, Ghana and Mali.

All the patients provided signed informed consent prior participation. Principle Investigator, statisticians and researchers did not have physical contact with the patients. All statistical analyses are reported previously [7]. The primary study was approved by Health Research Ethics Committee (HREC) of Stellenbosch University (HREC Reference # S18/07/150 (PhD) [7]. Conceptual framework worked described in details in previous published study [7].

## Results

The results of 126 female BC patients with stages ranging from 0-III initially subjected to AIs and subsequently followed up for 24 months. Follow up visits were carried out at the commencement of the study, month 4, month 12 and month 24. Mean age of 61 years (SD = 7, 11; 95%CI: 60–62). Linear regression revealed that hs-CRP was associated with waist circumference (OR: 7.5; p = 0. 0116; 95%CI: 1.45 to 39.61), and BMI (OR: 2.15; p = 0.034, 95%CI: 1.02 to 4.56). Random effects model revealed that different BC treatment strategies did not have effects on hs-CRP, BMI after 24 follow up. In contrast, there was stronger statistically association between BMI and homocysteine (p = 0.021, 95%CI: 0.0083 to 0.1029), weight and total body fat were strongly associated after 24 months follow up using multiple imputation data. Hs-CRP was associated with BMI (p = 0.000), and hs-CRP was associated with other inflammatory markers such as calcium (p = 0.021, 95%CI: 0.0083 to 0.1029), phosphate (p = 0.039, 95%CI: 0.0083 to 0.1029), and ferritin (p = 0.002, 95%CI: 0.0199 to 0.084). There was statistically significant variation between BMI, TBF, weight, hs-CRP, homocysteine, ferritin and calcium between baseline and after 24 months follow up. HyBeacon® probe technology at POC for AI-associated adverse events was cost-effective in Africa while adjunct to standard practice. Table 1 summarizes the impact of AIs on markers of inflammation at baseline and study endpoint.

**Table 1:** The effects of AIs on BMI and hs-CRP after 24 months of follow-up

Aromatase inhibitors at month 24	Coef	P-value	95% CI
TBF	0.119	0.0001	0.070 to 0.160
Weight	0.353	0.001	0.340 to 0.360
Hs-CRP	0.006	0.113	-0.001 to 0.014
Homocysteine	0.055	0.021	0.008 to 0.102
Phosphate	13.256	0.056	-0.364 to 26.878
Calcium	-35.945	0.017	-65.530 to -6.359
Phosphate	0.928	0.039	0.0470 to 1.809
Ferritin	0.052	0.002	0.019 to 0.084
BMI	0.835636	0.001	0.502 to 1.168

Legends BMI = body mass index, coef = coefficient, pth = phosphate, hs CRP = high sensitivity C reactive protein

The appropriate pathways for implementation of POC testing in postmenopausal breast cancer survivors need further investigation in different clinical settings with real data for external validity. The barriers for implementation of POCT application among six African countries for diagnosis of breast cancer included governance issues, insufficient awareness and insufficient trainings, lack of lab equipment’s, insufficient funding’s and ethical guidance issues for conducting genetic testing’s in African context. Table 2 summarizes the cross sectional data from 6 African countries focused on knowledge and barriers for implementation of POCT in Africa.

**Table 2:** Breast Cancer diagnosis and ParaDNA feasibility in African setting

Questionnaire	Number(N) = 50	Percentage (%)
**Knowledge on diagnosis of BC**		
Sanger sequencing Microscopy/histology Nanopore sequencing BRCA1/2 MammaPrint Breast ultrasound Mammogram/MRI TaqMan genotyping	20/50 37/50 24/50 5/50 15/50 22/50 4/50	40% 74% 48% 10% 30% 44% 8%
**Knowledge on ParaDNA genotyping**		
Benefit of ParaDNA/Sanger Identification of BRCA1/2 Prediction of CVD/comorbidities Turnaround within 75 min Easy to perform Can be cost effective/Sanger Automated DNA extraction, PCR and sequencing Can be used by non-specialized person	21/50 20/50 6/50 16/50 15/50 5/50 7/50 25/50	42% 40% 12% 32% 30% 10% 14% 50%
**Barriers to ParaDNA implementation**		
Limited accessibility Knowledge gaps Governance issues Lack of molecular lab for cancers Lack of trainings and awareness Lack of policy on point of care genetic testing’s Ethics and law regarding genetic testing’s	2/50 33/50 23/50 11/50 20/50 20/50 8/50	4% 66% 46% 22% 40% 40% 16%
**Application of POCT in diagnostic of COVID-19**		
Yes No	21/50 29/50	42% 68%
**Recommendations**		
Training and equipment Providing molecular lab and devices	11/50 39/50	22% 78%

However, there is a lack of data on costs and effects of POCT in breast cancer patients and associated comorbidities using real patient data in the rest Africa for external validation.

This study revealed that hs-CRP and BMI are predictors of CVD-related adverse events in obese postmenopausal patients. Calcium, phosphate, homocysteine, and ferritin should also be incorporated in POCT. There were statistically significant variations in TBF, weight, hs-CRP, BMI, homocysteine, ferritin, and calcium between baseline and after 24 months of follow-up. This is the first study assessing the feasibility of POCT in identifying unifying risk factors associated with breast cancer therapy side effects in African settings, including barriers for implementation of personalized medicines were documented in five African countries [7].

Both mathematical modeling and clinical data identified that POC genotyping may be cost effective in clinical African settings. A POCT must ideally be cost-effective, rapid, functional without excessive prior-processing of samples, highly sensitive to enable detection of BC and associated comorbidities for differential diagnosis, and specific to prevent therapy side effects, underdiagnoses, or over -diagnosis. POCT must facilitate “self-use” or use by a general practitioners or nurses in primary care settings from low limited settings of Africa. Results must be returned in a timely manner to initiate treatment as soon as possible, which ultimately leads to the enhancement of the patient’s wellbeing. The knowledge assessment from HCW from African settings on the implementation of POCT testing revealed that low level knowledge on diagnosis in breast cancer patients among 6 countries of Africa. The most predominated barriers for knowledge gaps identified including limited accessibility to molecular laboratories, knowledge gaps governance issues, lack of molecular laboratory or BC diagnosis, insufficient trainings and ParaDNA equipment, lack of policy on point of care genetic testing’s, ethical concerns regarding genetic testing’s and limited scientists in molecular diagnosis and public health awareness on breast cancer risk factors, diagnosis and management. Comparison of POCT genotyping showed similarity with other studies.

Implementation of POC genetic testing based on the current level of evidence will facilitate differential diagnosis and reduce the waiting time and costs associated with payment of scientists, while waiting for negative results from Sanger sequencing [7]. NCD screening may occur in clinical settings and remote communities to guide the therapeutics, especially considering the fact that there is a scarcity of cancer diagnosis in various African settings. The other aspect to consider is policy on the genetic testing in Africa. Policy guidelines, acceptability, and feasibility of hybean probe genotyping need further studies, since Sanger sequencing is not yet feasible for the unique characteristics of African clinical settings.

In conclusion, this study revealed that hs-CRP, BMI, calcium, phosphate, homocysteine, and ferritin should be incorporated in PSGT. POCT can be used with PSGT strategy to facilitate early diagnosis and prediction of side effects associated in African settings. The barriers for implementation of POCT application among six African countries for diagnosis of breast cancer included governance issues, insufficient awareness on breast cancer risks, insufficient trainings, lack of lab equipment’s, insufficient funding’s and ethical guidance issues for conducting genetic testing’s in African context.

## Limitations

Mathematical equations involving decision trees maybe confusing, if at all possible, and require numerous simplifying assumptions than real life events. Genotyping test has not yet been used in postmenopausal breast cancer patients on AIs to identify the high penetrant genes in many African settings. Although, its applicability to assess the cost-effectiveness of genotype-guided dosing in CVD patients may differ according to different settings and population diversity [7]. The characteristics of South African breast cancer survivors may not be similar to those studies used in the current computations. Other studies should be conducted at large public health scales to consider a disruptive commissioning model that includes reimbursement and other incentives to affect the large-scale adoption of suitable multi-array POC devices, including specified CYP19A1 alleles with the potential to reduce cost and maximize patient benefits [8–11].

## Data Availability

Available upon request on https://scholar.sun.ac.za/handle/10019.1/100763.

## Abbreviations

AIs: Aromatase inhibitor.
BC: Breast Cancer.
BCS: Breast cancer survivors.
CVD: Cardiovascular diseases.
HCW: Healthcare workers.
ICER: Incremental cost effectiveness ratio.
NPV: Negative predictive value.
PPV: Positive predictive value.
POCT: Point of care technology.
POC: Point of Care.
